# Mitophagy and Oxidative Stress in Cancer and Aging: Focus on Sirtuins and Nanomaterials

**DOI:** 10.1155/2019/6387357

**Published:** 2019-05-09

**Authors:** Enza Vernucci, Carlo Tomino, Francesca Molinari, Dolores Limongi, Michele Aventaggiato, Luigi Sansone, Marco Tafani, Matteo A. Russo

**Affiliations:** ^1^Department of Cardiovascular, Nephrologic, Anesthesiologic and Geriatric Sciences, Sapienza University of Rome, Viale Regina Elena 324, 00161 Rome, Italy; ^2^IRCCS San Raffaele, Scientific Direction, Via Val Cannuta 247, 00166 Rome, Italy; ^3^Department of Experimental Medicine, Sapienza University of Rome, Viale Regina Elena 324, 00161 Rome, Italy; ^4^IRCCS San Raffaele Pisana, Department of Human Sciences and Promotion of the Quality of Life, San Raffaele Roma Open University, Via Val Cannuta 247, 00166 Rome, Italy; ^5^Department of Experimental Medicine, Sapienza University of Rome, Viale Regina Elena 324, 00161 Rome, Italy; ^6^Department of Cellular and Molecular Pathology, IRCCS San Raffaele, Via Val Cannuta 247, 00166 Rome, Italy; ^7^Department of Experimental Medicine, Sapienza University of Rome, Viale Regina Elena 324, 00161 Rome, Italy; ^8^MEBIC Consortium, San Raffaele Rome Open University, Via Val Cannuta 247, 00166 Rome, Italy

## Abstract

Mitochondria are the cellular center of energy production and of several important metabolic processes. Mitochondrion health is maintained with a substantial intervention of mitophagy, a process of macroautophagy that degrades selectively dysfunctional and irreversibly damaged organelles. Because of its crucial duty, alteration in mitophagy can cause functional and structural adjustment in the mitochondria, changes in energy production, loss of cellular adaptation, and cell death. In this review, we discuss the dual role that mitophagy plays in cancer and age-related pathologies, as a consequence of oxidative stress, evidencing the triggering stimuli and mechanisms and suggesting the molecular targets for its therapeutic control. Finally, a section has been dedicated to the interplay between mitophagy and therapies using nanoparticles that are the new frontier for a direct and less invasive strategy.

## 1. Mitophagy

**Figure 1:**
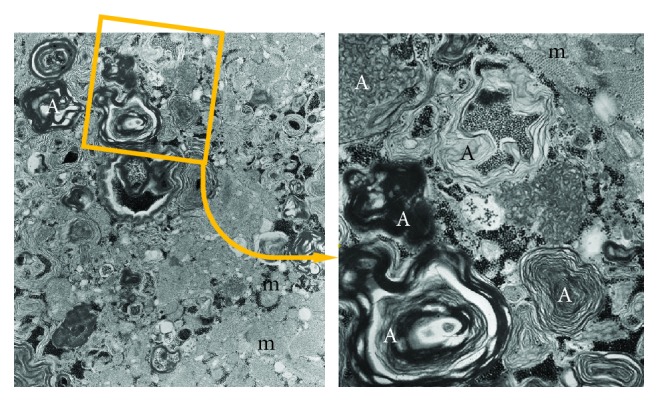
Autophagosomes accumulate into myocardiocytes due to the presence of chloroquine which blocks the fusion between primary lysosomes and autophagosomes. (a) Large autophagosomes (A) with different cytoplasmic components, mainly degraded lipid membranes, and glycogen. (b) Magnification of the detail indicated in the square. m: mitochondria.

**Figure 2:**
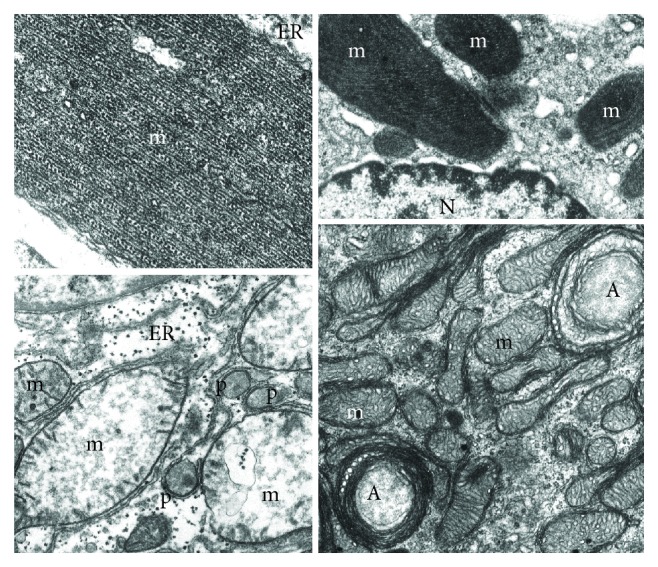
Mitochondria (m) have been damaged by different physiopathological conditions. (a) Mutated, misfolded, and fibrillary polymerized mitochondrial proteins may accumulate into the matrix giving rise to giant mitochondria with paracrystalline inclusions. They appear surrounded by endoplasmic reticulum (ER) membranes, indicating a process of segregation for autophagocytosis. (b) Antibiotics affecting bacterial protein synthesis may interfere with mitochondrial protein synthesis producing enlarged mitochondria (m) with paracrystalline inclusions and bizarre shape. Also, in this case, the close interaction with endoplasmic reticulum membranes suggests a process of segregation for mitophagy (N: nucleus). (c) Swollen liver mitochondria (m) after 3 hours of ischemia: they show a number of pathological changes: volume increase, dishomogeneous electron-clear and sometimes vacuolized matrix, fragmented cristae, and sometimes interrupted outer membrane. Indeed, they appear, together with apparently intact peroxisomes (p), surrounded by endoplasmic reticulum (ER) which indicates the autophagocytic process. (d) Mitochondria (m) from the glomerular zone of a suprarenal cortex which has been intensively stimulated by ACTH. The consequent hypertrophy includes also an increase of mitochondrial growth (number and volume) and an accelerated turnover as suggested by the increased mitophagy (A).

The cellular process that involves the degradation of aged and/or damaged mitochondria by autophagy is known as “mitophagy” [1]. Mitophagy is a physiological mechanism requested for mitochondrion turnover [2] and cell maintenance and for responding to novel energetic requirements [3]. In *Caenorhabditis elegans*, mitophagy is required in the oocytes for removing the mitochondria (and other organelles) of paternal origin thanks to the interaction of ALLO1 and IKKE-1 that drive organelle clearance [4]. Excessive, defective, or inappropriate mitophagy is responsible for cellular damage and death [5]. Abnormal mitophagy may be primary due to primitive mutations of genes involved in its mechanisms or secondary to an excessive mitochondrial damage, such as mitochondrial depolarizing stimuli, hypoxia, toxic agents (Table 1), radiations, and accumulation of mtDNA mutations (Figures 1 and 2).

**Figure 3:**
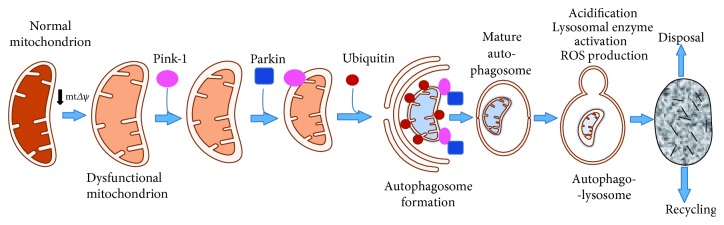
Sequence and molecular details of the selective mitophagy. When mitochondria undergo decrease in membrane potential (or different stress stimuli), PINK1, localized on the mitochondrial membrane, recruits Parkin that polyubiquitinates MOM protein and induces the autophagosome formation. Then, lysosome fuses with the autophagosome (autophagolysosome) and the degraded material can be recycled or disposed.

Mitophagy is a specific form of macroautophagy which occurs as a multistep process, including (1) the selection/segregation of these organelles in a vacuole (*autophagosome*), (2) the fusion with a lysosome (*autophagolysosome*) [16], (3) the acidification of internal microenvironment [17, 18], (4) the activation of lysosomal acidic enzymes and oxidative metabolism [19], (5) the degradation of the content, and (6) the recycling and disposal of the final products (Figure 3). Only in the last few years the mechanisms and molecules involved in these different phases of mitophagy have been identified, although several questions are still unanswered.

When aged or irreversibly damaged mitochondria are destined to disposal, they are marked on the mitochondrial outer membrane with receptors that can interact *directly* with their *countereceptors or ligands* localized on endoplasmic reticulum membranes (Table 2) or *indirectly*, through a ubiquitin-mediated process involving Parkin (E3 ubiquitin ligase) and PINK1 (PTEN-induced kinase 1), known to interact with Beclin 1 [20] and several other proteins [21].

**Figure 4:**
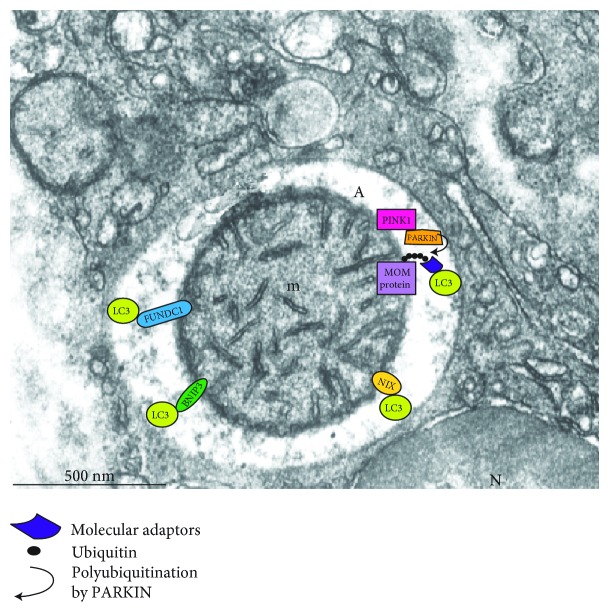
Electron micrograph of a mitophagic vacuole (A) containing a well-preserved mitochondrion (m). Molecules involved in selective mitophagy are indicated.

In both cases, a similar multistep process occurs, as outlined above:

*Macroautophagocytosis* is the receptor-mediated selection and encapsulation/engulfment of organelles by the endoplasmic reticulum membranes, forming a vacuole, probably with a zip interaction between the two membranes (Figure 4) [35].

The best known membrane receptors (Table 2) implicated in mitophagy are NIX/BNIP3L, BNIP3, and FUNDC1 linked to hypoxia-induced mitophagy, BCL2L13, AMBRA1, SMURF1, FKBP8, and PHB2 (prohibitin 2). Recently, NLRX1 is a Nod-like receptor family member used by *L. monocytogenes* to induce mitophagy to survive inside host organisms [34]. All of them, through their LC3-interacting regions (LIR), can recruit specific proteins and start encapsulation inside the autophagosome. FUNDC1, interestingly, interacts with OPI1, DNMIL, and LC3 according to its phosphorylation status. In fact, it has been reported that two kinases (SRC and CK2) and the phosphatase PGAM5 through phosphorylation and dephosphorylation can determine FUNDC1 “interactome” [36]. FUNDC1 interacting with HSC70 plays a pivotal role also in the translocation to the mitochondria of unfolded cytosolic proteins for degradation by LONP1 or for nonaggresomal mitochondrion-associated protein aggregate (MAPA) formation that will be eliminated by autophagy [37]. The interaction between PINK1 and Parkin, instead, is fundamental for mitochondrial quality check [38]. Parkin can be directly phosphorylated by PINK1 on serine 65 [39], or PINK1, through the ubiquitin phosphorylation on serine 65, can recruit and activate Parkin in an indirect way [40]. It has been published that a new PTEN isoform, PTEN-L (PTEN-long), can interfere with Parkin translocation and, thanks to its dephosphorylase activity, can diminish Parkin and ubiquitin phosphorylation acting as a mitophagic inhibitor [41]. When PINK1 accumulates on the mitochondrial outer membrane (MOM) following a decrease in mitochondrial membrane potential [42], it undergoes autoactivation and recruits Parkin [43] that, in turn, polyubiquitylates several proteins located on the MOM and starts the fission process [44, 45]. This polyubiquitylation process can be inhibited by USP30, a deubiquitinase found in neurons that, removing ubiquitins transferred by Parkin on damaged mitochondrial proteins, blocks Parkin-mediated mitophagy [46]. Parkin is not the only E3 ligase involved in mitophagy; recently, it has been shown that during selenite-induced mitophagy, ULK1 translocates to the mitochondria where it is ubiquitinated by MUL1 and PARK2 and FUNDC1-independent mechanism [47]. Polyubiquitylated MOM proteins mobilize many adapter proteins such as TAX1BP1, NBR1, p62, NDP52, and OPTN that are important for the PINK1/Parkin-mediated mitophagy and for the interaction between polyubiquitin chains and Atg8-like proteins [48, 49] that drive the autophagosomal-lysosomal pathway [1].

The interaction of a phagosome with a lysosome is carried out with the cooperation of many different protein complexes, including a transport system (rab/microtubules), a fusion system (SNARE proteins) responsible for the fusion of the two membranes, and a tethering system which facilitate the specificity of interaction and the rapid sealing of the two opposing membranes. The detailed molecular mechanisms have been extensively reviewed by Nakamura and Yoshimori [50].

The *acidification* of the internal milieu of the phagolysosome depends on the strong activity of the “vacuolar” ATPase (V-ATPase). This protein complex acidifies the lumen of many different intracellular compartments (including lysosomes, phagosomes, and autophagosomes) by transporting protons against a gradient from a cytosol into the lumen of the vacuole at the expense of ATP hydrolysis. The low pH is required for lysosomal enzyme activity and for further demolition of the phagosomal content [18, 50].

The a*ctivation* of lysosomal degrading enzymes is accompanied by oxidative metabolism burst [19]. This leads to a further damage of the content, with an easier demolition, fragmentation, and digestion of the mitochondrion and other cytosolic content, and recycling or extracellular disposal of the final products.

Many autophagy-related proteins especially receptors and their interacting ligands are also known as mitophagosomal marker proteins, coded by an autophagy-specific battery of genes (Table 3).

## 2. Mitophagy in Cancer

Mitophagy has been linked with several physiological functions and human pathologies like neurodegenerative disease [59–61], type 2 diabetes [62], cardiac defects [63], and tumor [64]. The connection between tumor and mitophagy is complex and controversial and probably is connected to oxidative metabolism and energy homeostasis. Mitochondria are the primary site for ATP production, but they are also the place where reactive oxygen species (ROS) production and glucose metabolism occur. Generally, tumors undergo metabolic reprogramming to gain advantages with respect to surrounding cells [65, 66]. Several studies have shown that KRAS plays a pivotal role in a variety of cancers promoting readjustment of cell metabolism [67]. TBK1, a mitophagy effector, seemed to be involved in KRAS activity [68]. It is overexpressed in different kinds of malignancies such as lung, breast, and colon cancer [69], and it is requested for KRAS-driven cell transformation. TBK1 null cells infected with retrovirus encoding for KRAS were unable to proliferate and survive [70]. In KRAS-mediated lung tumors, the depletion of Atg5 or Atg7, two mitophagic effectors involved in LC3/GABARAP lipidation, has induced a reduction in tumor burden and an increase in survival compared to the counterpart even if malignancies present a faster tumor-initiation stage [71, 72]. Parkin also plays an important role in cellular metabolism balance. It has been discovered that Parkin is a p53 target gene and contributes to p53 glucose metabolism regulation and mitochondrial respiration [73]. In fact, Parkin can mediate the p53 reduction of the Warburg effect decreasing cellular glucose uptake and lactate release [74]. Parkin-mediated glycolysis reduction can also be performed through PKM2 regulation. It has been shown that Parkin can ubiquitinate this isoform of pyruvate kinase and can reduce its enzymatic activity [75]. But the role of Parkin and glycolysis regulation is contentious. In fact, Parkin can positively regulate the expression of PDHA1 reducing, in this way, mitochondrial oxidative phosphorylation and increasing the glycolytic pathway [74, 76].1Because the Warburg effect represents a hallmark of cancer cells that, using aerobic glycolysis, try to sustain the energetic demand, it is clear that mitophagy and cancer can be strongly related. Hypoxia-inducible factor 1 (HIF-1), one of the major drivers of metabolic rewiring in cancer, is involved in mitochondrial autophagy. Through the induction of BNIP3, HIF-1 triggers mitophagy as a metabolic shift due to hypoxia and prevents ROS increase and cell death [26]. Lipid metabolism is an important biochemical step in tumorigenic cells that can either increase endogenous synthesis or promote lipid uptake to face the demand for biomass [77]. Parkin, in turn, can stabilize CD36, a fatty acid transporter, through a ubiquitin-mediated process and regulate lipid transport [78].

Genetic instability is known to be a common factor in a wide range of cancer [79]. Modification in copy number, amplifications, and mutations in genes involved in mitophagy are frequent in several tumors, and this raises the possibility that all these alterations provide an advantage for tumor growth. In colorectal cancer, for example, Parkin is deleted in 25% of cases [80] and 33% of patients revealed the heterozygous loss of the gene above [81]. A tumor suppressive role of Parkin is also detected in breast cancer where the blockage of mitophagy influences tumor progression [82, 83] and in hepatocellular carcinoma where mouse knockout for this gene showed enhanced growth of hepatic tumors [84]. Looking at the glioblastoma, the role of Parkin seems to be under debate. The TCGA database showed that a quarter of patients affected by glioblastoma exhibited a partial or total loss of *PARK2* (gene encoding for Parkin) [85] but has also demonstrated that silencing of Parkin can be related to the arrest in tumor growth through the cooperation with the Notch signaling pathway [86]. PINK1 resulted to be altered in several cancers such as ovarian cancer, glioblastoma, and neuroblastoma [87–89] and the same came from BNIP3 screening. BNIP3 appeared to be lost in invasive breast cancer [90] and in almost 60% of pancreatic cancer patients where it correlated with poor survival [91]. In fact, pancreatic cancer cells showed hypermethylation of the BNIP3 promoter that prevented HIF-1 binding and the subsequent activation of mitophagy that restrained mitochondrial mass and ROS production [92]. The correlation between BNIP3 and tumor progression/metastasis formation is investigated in triple negative breast cancer where BNIP3 null tumor cells enhanced ROS formation that, in turn, led to HIF1 alpha activation and invasive phenotype [93]. Also, FUNDC1 expression has been correlated to the initiation and progression of hepatocarcinogenesis. In a mouse model, the hepatocyte-specific knockout of FUNDC1 revealed a blockage in mitophagy characterized by an increase in mtDNA release and inflammasome activation that contributes to tumorigenesis [94]. It is an arduous task to predict the role that mitophagy has on cancer cells because it depends on different factors like cancer type, cancer stage, genetic background [95], and equilibrium between cellular demand and availability; according to the scenario, mitophagic alterations can have a dual role acting as cancer suppressors like during Atg5 or Atg7 depletion or promoters like BNIP3, FUNDC1, and PINK1 deficiency.

The connection between the immune system and cancer has been widely studied in the last decades due to the capability to be both enemies and allies [94]. In some cancers, the presence of a particular kind of immune cells in a tumor microenvironment can help to understand patient outcome. It is the case of colorectal cancer where CD8^+^ T cells infiltrating tumor are associated with prolonged survival and a better prognosis than T helper 17 cells [95]. It has been reported that increased mitophagy, in intestinal epithelial cells, enhanced lysosomal membrane permeabilization and stimulated MHC I presentation and CD8^+^ T cell activation showing the intrinsic antitumor function of mitophagy [95]. Also in lung cancer, autophagy/mitophagy is coupled to immunosurveillance [71]. Kras^G12D/+^ Atg5^fl/fl^ mice were characterized by the presence of a remarkable number of regulatory T cells (Tregs) known to suppress the immune system and responsible for the improved tumor initiation in these mice compared to the control group [71].

A plethora of genes that are not direct effectors of mitophagy resulted to be altered in malignancies. Their role has been studied, and a hypothetical correlation with mitochondrial autophagy has been developed. This is the case of YAP (yes-association protein), a downstream effector of the Hippo signaling pathway, and Bif 1 (a member of endophilin family proteins) that resulted to be altered in a variety of cancers [96–100]. Lately, the role of YAP in activating mitophagy via the SIRT1/Mfn2 axis and its contribution in migration and viability in gastric cancer have been highlighted. In fact, through the preservation of SIRT1 activity, YAP can sustain the Mfn2-mediated mitophagy, reduce ROS production, and increase ATP generation, involved in cell migration support [101]. Bif-1, conversely, is crucial for mitophagy because it regulates the maturation of autophagosome. The Bif-1 haploinsufficiency caused the accumulation of immature autophagosomes leading to damaged mitochondria and increased ROS production that has promoted MYC-driven lymphomagenesis [102].

Mitophagy seems to be also involved in cancer-induced cachexia where the analysis of the transcriptome dataset revealed the upregulation of genes involved in phagophore elongation and maturation that characterize the latest step of mitophagy [103]. An increase in the activity of lysosomal proteases has been reported in the cachectic muscle in tumor-bearing mice [104]. Furthermore, the skeletal muscles from cancer patients and mouse models have shown an increase of mitophagic parameters [105, 106]. The mitochondrial involvement in cancer-induced cachexia remains to be elucidated, but this can open the opportunity to new therapeutic strategies to reduce muscle wasting that impairs further the quality of life of cancer patients.

Because of the dual role the mitophagy has in cancer depending on different situations and cell types, a variety of studies have been developed to understand the impact the mitophagy has on chemotherapy. The efficiency of damaged mitochondrial clearance can mediate drug resistance in tumor cells [107] since the evidence indicating that chemotherapeutic drugs can induce mitochondrial dysfunction and ROS production [108]. Different agents as ceramide and ceramide analogs, causing lethal mitophagy, have been used in cancer therapy to induce cancer cell death [14, 109] and decrease drug resistance [110]. On the other hand, mitophagy inhibitors can enhance chemotherapeutic sensitivity. Doxorubicin, salinomycin, and UNBS1450, drugs used for cancer treatment, resulted to be more effective during mitophagy inhibition [107, 111, 112]. Understanding mitophagy behavior during cancer development and growth can help to discern if mitochondrial autophagy acts as tumor promoter or suppressor. Inhibiting or activating mitophagy can be crucial for the therapy success. All these reasons highlight the relevance of this process and drive researchers to develop new drugs to regulate it.

## 3. Mitophagy in Aging

Mitochondria are the energy center of cells. Mitochondrial maintenance is a prerequisite for the homeostasis of cells and organisms. The equilibrium between mitochondrial biogenesis and mitochondrial removal is crucial for a healthy system. Several studies focused on the evidence the accumulation of nonfunctional mitochondria, therefore defective mitophagy, may have with aging and age-related disorders [113]. Aging is a process known to be regulated by a preserved signaling pathway. Alterations in those pathways along with perturbations in mitochondrial functions and efficiency lead to cellular and tissue degeneration [114]. AMPK is a regulator of energy metabolism [115] and has been associated with mitophagy [116]. AMPK, activated by various stress stimuli, can promote autophagy and mitochondrial autophagy through mTOR inhibition [117] or ULK1 activation [118]. Thus, AMPK stimulation could represent an option to fight age-related diseases and prolong survival. In fact, it has been reported that mTOR downregulation is involved in extended lifespan in Drosophila and mice [119]. AMPK can also interact with Sirt1, a member of the sirtuin family known to play a pivotal role in metabolism and aging [120]. SIRT1, along with other sirtuins, induced an alteration in the NAD+/NADH ratio that decreased during aging in a variety of organs [121, 122]. Sirt1 has been associated with mitophagy starting from some evidence such as excessive mitochondrial damage in Sirt1 knockout mice [123], deregulation of Pink1, and impaired mitochondrial autophagy in NAD+-Sirt1-Pgc-1 alpha axis alteration [124].

The aging process is one of the principal risks for neurodegenerative diseases like Alzheimer (AD) and Parkinson's disease (PD), and Fuchs Endothelial Corneal Dystrophy (FECD). AD is the most common neurodegenerative disorder and is characterized by neurofibrillary tangles and plaques containing amyloid-*β* peptide [125]. Numerous evidences linked alterations in mitochondrial quality control with AD [126]. Using a triple transgenic mouse with perturbations in APP (amyloid beta precursor protein), Tau and PS1 have shown that Parkin ubiquitinated A*β* and reduced its levels stimulating its degradation in a Beclin-dependent manner [127]. Experiments in a variety of mouse models of AD showed that the administration of NAD+ precursors could reduce A*β* plaques and the cognitive decline [128, 129]. The authors attributed the reduction of AD phenotype to the ability of NAD+ to increase Sirt1 activity as discussed above. Through the upregulation of proteins involved in autophagy/mitophagy [6] or the FOXO3-NIX axis [51] or the interaction with PGC-1*α* and Parkin [130], NAD+/SIRT1 can improve mitophagic activity and neuronal survival [131]. Several pieces of evidence link Parkinson to mitophagy; first of all, the mitochondria are defective (alteration in complex I of electron transport chain) [132]; second, Parkin and PINK 1 resulted mutated in PD patients [133]; and third, these mutations caused perturbations in mitochondria clearance [134]. In *Drosophila melanogaster*, knockouts of PINK 1 and Parkin have induced impaired mitophagy leading to defective dopaminergic neurons and locomotion [135]. Unfortunately, when these genes were manipulated in mice, researchers did not obtain the same phenotype [133]. Mice null for Parkin were bred with mice constituted by mutations in mtDNA polymerase; the offspring showed degeneration of dopaminergic neurons implying that the inability to repair and remove mutated mtDNA through mitophagy was linked to Parkinson-like pathologies [136]. In sporadic and familiar cases of PD, a decrease in Miro (MOM protein removed right before the beginning of mitochondrial clearance) has been reported [137]. Leucine-rich repeat kinase 2 (LRRK2), PINK 1, and Parkin were the three genes highly mutated in those patients. LRRK2 loss of function was unable to interact with Miro, and the mitophagy initiation was delayed causing neurodegeneration [137]. A recent study showed that USP30, a mitochondrial deubiquitinase, antagonized Parkin and PINK 1 activities. Thus, USP30 removed ubiquitin from damaged mitochondrial proteins and inhibited mitophagy. USP30 knockout in dopaminergic neurons can improve mitochondrial clearance and locomotion rescuing the defective mitophagy caused by mutations in Parkin and PINK 1. In this scenario, USP30 inhibitors can represent a potential target for PD [46]. FEDC is the most common degeneration of corneal endothelial cells during aging. The authors demonstrated that induction of mitophagy was involved in the reduction of mitochondrial mass and functional mitochondria. The analysis of tissues from FEDC patients revealed autophagic structures containing mitochondria that were indicative of an upregulated auto/mitophagy. To validate the role of mitophagy in FEDC, a decline in Mfn2, an important fusion protein, was detected confirming that the fusion capacity was lost and the fission-mediated mitophagy prevailed [138]. Despite the role the mitophagy plays in age-mediated diseases, as we know, mitochondria are the place where ROS are produced [139]. ROS are known to induce mutations into the nuclear DNA and mtDNA. The repair machinery in the mitochondria is less efficient compared to the one inside the nucleus, and this impairs the synthesis of enzymes involved in oxidative phosphorylation leading to an energetic failure. Thus, in this scenario, mitophagy can be seen as a mechanism to prevent the accumulation of mtDNA mutations and the development of age-associated diseases like cancer, diabetes, and neurodegenerative diseases.

The correlation between mitophagy and age-related muscle wasting and sarcopenia has been under debate in the last decades. In a muscle, autophagy declines with aging contributing to tissue degeneration [140, 141]. Lately, it has been documented that aged skeletal muscle is characterized by an increase of autophagic/mitophagic proteins [142, 143]. A very recent study showed that, using a model of aging, the Fisher 344 Brown Norway Hybrid rat, an enhancement in mitophagy flux and increasing in mitophagy receptors was detected in an aged muscle. This observation was by the reduced presence of organelles in those muscles [144]. Also, lysosomes, involved in autophagosomal content degradation, decreased in muscle during aging [143]. Not much is known about the effect of exercise on mitophagy in an aged muscle. After chronic contractile activity (CCA), muscles were characterized by an increase in the mitochondria even if it was less evident than the younger counterpart. CCA also induced a reduction of TFEB expression, the primary regulator of lysosomal biogenesis, contributing, perhaps, to the mitochondrial asset in an aged muscle [144]. Using denervation and unilateral hind limb immobilization as a model of muscle disuse, it has been demonstrated that mitochondrial autophagy was increased [145, 146] and the silencing of Parkin was sufficient to avoid mitochondria clearance in the soleus muscle preventing the maintenance of healthy organelles [147].

Aging is also known to increase the liver sensitivity to ischemia/reperfusion (I/R) via induction of mitochondrial damage and malfunction [148]. Thinking about the increasing possibility to use elderly patients as potential liver donors, a recent study highlighted the role the defective mitophagy plays in this process. The authors demonstrated that mitophagy protected the liver from I/R injury, in fact, decreasing in Parkin and Atg5 detected in old mice's liver during hepatic I/R injury. Using salubrinal, an inhibitor of the protein phosphatase PP1 involved in EIF2*α* dephosphorylation has obtained an induction of Parkin and mitophagy after reperfusion enhancing the response to I/R injury [149].

## 4. Mitophagy and Sirtuins

Sirtuins represent a new class of proteins that, recently, has been deeply involved in controlling several pathways linked to mitophagy. In fact, sirtuins are a class of seven (SIRT1-7) NAD+-dependent deacylases with ever-growing intracellular targets: histones, transcription factors, metabolic enzymes, structural proteins, etc. [150]. Due to the NAD+ dependence, sirtuins can sense the metabolic status of the cell and increase or decrease their activity in order to maintain homeostasis [151]. In fact, sirtuin activity increases during caloric restriction or in the presence of natural activators such as resveratrol [152], curcumin [153], and piceatannol [154]. On the contrary, sirtuin activity decreases during high-fat diet [155, 156]. Moreover, sirtuin activity is fundamental for the cellular response to stresses such as hypoxia, exercise, and ROS accumulation [157–160].

Recently, sirtuin expression and activity have been linked, either directly or indirectly, to the control of mitophagy during diverse pathological conditions such as cancer, neurodegeneration, diabetes, and sepsis as well as during aging, chemotherapy toxicity, and starvation [161–166].

However, up to date, an involvement in mitophagy control has been demonstrated only for SIRT1, SIRT2, and the mitochondrial sirtuins SIRT3, SIRT4, and SIRT5.

In particular, loss of SIRT1 has been associated to decreased mitophagy and delayed PARK2 accumulation in the mitochondria in prostatic intraepithelial neoplasia. Loss of SIRT1, observed in the luminal epithelium of human prostate cancer, determined an accumulation of ROS and an inactivation of SOD2 an effect that, in turn, caused a deregulation of mitophagy, and it was at the basis of the worsening of the patient outcome [166]. Altered mitophagy due to SIRT1 decrease has also been demonstrated in Xeroderma pigmentosum group A (XPA) as well as ataxia-telangiectasia (AT) and Cockayne syndrome (CS), all associated with neurodegeneration and cancer. In this case, mitophagy deregulation is due to the activation of PARP1 that, in turn, caused the decrease of NAD+-SIRT1-PGC-1a-UCP2 axis. In fact, both PARP1 inhibitor and NAD+ precursor can rescue XPA cells and increase lifespan in xpa-1 nematodes [124]. Low levels of MKK kinase associated with high levels of Sirt1 diminished lethality of sepsis in mice through an activation of mitophagy removal of damaged mitochondria and activation of PGC-1a-induced mitochondriogenesis [167]. In cancer, SIRT1 activation by a new compound increased autophagy/mitophagy thereby reducing glioblastoma growth in vitro and in vivo [168]. However, mitophagy activation can also increase tumor survival as observed in gastric cancer where survival and migration of cancer cells were maintained by YAP-Hippo-Sirt1-MFN2 activation of mitophagy [101]. SIRT1 upregulation increased mitophagy in an infarcted heart following liraglutide treatment. Such an effect is achieved through the SIRT1-driven increase of Parkin leading to mitophagy activation [169]. Another important aspect that must be considered is that different sirtuins can interact with each other to regulate mitophagy. In fact, SIRT1 can deacetylate and activate SIRT3 that, in turn, controls mitochondrial health also by regulating mitophagy. In particular, in obese and old mice, low levels of Sirt1 are accompanied by hyperacetylated Sirt3 and dysfunctional mitochondria [170].

The involvement of SIRT2 in mitophagy control has been demonstrated in the brains of Sirt2 knockout mice that revealed a dysregulation of mitochondrial proteins and mitophagy with the appearance of small and irregular mitochondria [165]. Moreover, SIRT2 ablation following vincristine treatment activated mitophagy and apoptotic cell death in the breast cancer cell line MDA-MB-231. In this situation, SIRT2 loss determined acetylation of HSP70 that, in turn, was responsible for mitophagy activation [171].

The role of SIRT3 in mitophagy regulation has been extensively documented in several pathologies. In fact, activation of SIRT3 was associated with induction of mitophagy to control mitochondrial homeostasis and remove dysfunctional mitochondria in mammalian cells. However, in the case of tumor cells, this may result in increased survival as demonstrated in glioma and breast cancer cell lines under hypoxia. In this situation, tumor cells increased SIRT3-driven mitophagy to counteract the damaging effects of oxygen reduction to a point that SIRT3 inhibition increased apoptotic cell death and ROS accumulation [172]. On the contrary, SIRT3-increased mitophagy was important for survival of myocardiocytes. In fact, Sirt3 KO worsened the development of diabetic cardiomyopathy (DCM) in a mouse model because of the impairment of the Foxo3A and Parkin pathway with reduction of mitophagy [173]. Similar results were obtained in aged hearts from Sirt3 KO mice that revealed a decrease in MnSOD, an increase in ROS, and an impairment of Parkin-mediated mitophagy [163]. These results suggested that a strategy is aimed at increasing SIRT3 expression and/or activity may ameliorate the outcome of cardiac pathologies linked to diabetes, aging, etc. It is worth noting that Sirt3 also activated mitophagy and cardiomyocyte survival in aged rats with stable myocardial infarction (MI). In particular, Sirt3 levels increased after short-term exercise in these rats with reduction of ROS and activation of mitophagy [174].

SIRT3 has also shown an important role in protecting hepatocytes during nonalcoholic fatty liver disease. In vivo studies have shown that Sirt3 overexpression increased Bnip3 level to activate mitophagy thereby maintaining functional mitochondria. Moreover, Bnip3 expression depends on the ERK-CREB pathway [161]. SIRT3 overexpression in amniotic fluid stem cell (AFSC) transplantation as a therapeutic strategy for diabetic nephropathy increased mitophagy and ameliorated the glucose metabolic parameters in vivo [162]. Therefore, SIRT3 overexpression or activation may be used to improve the outcome of stem cell therapy because of its prosurvival effects of these cells.

The less studied SIRT4 has been recently associated with mitophagy inhibition during aging. In fact, in aged fibroblasts, SIRT4 induction increased mitochondrial ROS production and associated with L-OPA1 to promote mitochondrial fusion. Such elongated mitochondria accumulate in aging fibroblast and are not removed by mitophagy thereby accelerating the aging process [164].

Similarly, to the other two mitochondrial sirtuins, also, SIRT5 has been linked to mitophagy. Inhibition of SIRT5 expression or activity in tumor cells or in myoblasts was accompanied by a reduction of BNIP3 and mitophagy. In these cells, SIRT5 interacted and inhibited mitochondrial glutaminase 1 impairing glutamine metabolism with reduction of ammonia levels. Ammonia reduction resulted in inhibition of autophagy and mitophagy [175]. Moreover, during starvation, SIRT5 targeted fission proteins to reduce their expression. This resulted in an elongation of mitochondria that cannot be removed by mitophagy. Therefore, SIRT5 helps to maintain mitochondria population during starvation [176].

In the case of the nuclear sirtuin SIRT6 and the nucleolar sirtuin SIRT7, no connection with mitophagy has been documented so far.

Finally, some important points arise from the scientific literature connecting sirtuins and mitophagy: (1) Mitochondrial sirtuins have crucial and opposing roles in regulating mitophagy. So far, SIRT3, the most abundant mitochondrial sirtuin, has always been linked to mitophagy activation. On the contrary, SIRT4 and SIRT5 have been linked to mitophagy inhibition. These opposing effects may be revealed important for the correct mitochondrial homeostasis. (2) There is a crosstalk between nuclear and mitochondrial sirtuins. In fact, SIRT1 can regulate SIRT3 expression by controlling the assembling of transcription factors on the SIRT3 promoter [177]. Moreover, SIRT1 can deacetylate and activate SIRT3 that can then efficiently regulate mitochondrial homeostasis through mitophagy [170]. (3) Sirtuins can control different metabolic pathways. In fact, emerging evidences indicate that mitochondrial sirtuins regulate not only glucose but also amino acid (glutamine) and fatty acid metabolism. The molecular details and importance of these global metabolic controls are still to be unraveled.

## 5. Mitophagy and ROS

The mitochondria are the main intracellular compartment responsible for reactive oxygen species (ROS) production. ROS generation represents a byproduct of oxidative phosphorylation, and although optimal ROS levels are essential for the regulation of physiological and biological mechanisms, ROS accumulation can alter macromolecules affecting cellular homeostasis and mitochondrial function [178]. A lot of studies suggested that damaged mitochondria can contribute to disease development and progression including NLRP3 inflammasome-related diseases. NLRP3 inflammasome enhanced innate immune defenses through proinflammatory cytokines, and its activation can be ROS-mediated [179]. A variety of stress stimuli can induce the production of ROS from the mitochondria [180]. It has been demonstrated that the blockage of complex I can increase ROS production that is positively correlated with proinflammatory cytokines like IL-1*β* in THP1 macrophages while the knockdown of NLRP3 did not cause the same phenomenon [181]. Dysregulated ROS-generating mitochondria are eliminated by mitophagy; therefore, inhibition of this process can increase ROS production and IL-1*β* secretion leading to chronic inflammatory diseases [181]. Mitophagy plays an important role also in hyperglycemia- (HG-) induced ROS overproduction; in fact, different studies showed how preventing mitochondrial dysfunction can reduce ROS concentration and endothelial damage in mice and patients affected with diabetes mellitus [182, 183]. Recently, it has emerged that mesenchymal stem cell (MSC) treatment can increase the expression of two mitophagic effectors, Parkin and Pink, reduce ROS production, and improve high-glucose-induced endothelial injury, consequently [184]. A cytoprotective role of ROS has been identified as well [185]. In fact, ROS generated during liver ischemia/reperfusion injury (IRI) in liver epithelial cells (LEC) have a regulatory role that involved the mitophagic pathway. It has been demonstrated, in *in vitro* and *in vivo* models, that ROS generation after IRI can lead to ATG7-dependent mitophagy and induce LEC survival [186]. ROS can also represent a mitophagy fuel [187, 188]. Studying the role of prooxidants, it has been observed that superoxide can drive Parkin-Pink-mediated mitophagy and this process required the p38 signaling pathway [188].

The main consequence under ER stress is the ROS production [26, 189, 190]. It has been demonstrated that miRNA-346 is induced under ER stress, and it is involved in autophagy/mitophagy activation to facilitate cell survival [191]. In fact, an increase of ROS during ER stress can be harmful to cancer cells [192]. In this scenario, miRNA-346 promoted mitophagy activation via GSK3*β* and reduced ROS preserving cell viability [191].

It is known that redox and O_2_ homeostasis are strictly connected. Hypoxia stimulates ROS increase leading to hypoxia-inducible factor-1*α* (HIF-1*α*) stabilization [193]. Reduced O_2_ concentration and increase of ROS also induce the inhibition of the prolyl hydroxylases responsible for HIF-1*α* degradation [194, 195]. HIF-1*α* activates mitophagy in a BNIP3-dependent manner producing a metabolic adaptation that allows cell survival and ROS decrease in a hypoxic environment in MEF cells [26], and BNIP3 absence in mammary tumor cells increases the Warburg effect, followed by ROS increase and tumor progression [93]. The same correlation has been evaluated in gastric cancer cells where mitophagy played a role in cancer aggressiveness [196]. In fact, mtROS production triggered by hypoxia was under the control of mitophagy and when this process was impaired, mtROS concentration increased and stabilized HIF-1*α* along with an aggressive phenotype [196].

## 6. Mitophagy and Nanoparticles

The term nanomaterial includes particles with a size range between 10 and 100 nm [197] and a shape that is directly correlated to biodistribution efficacy as carriers and interaction with the target tissues [198]. The most common are nanospheres and nanorods, but new nanocrystals have been developed [198]. Several studies have been promoted to evaluate the effect of nanoparticles on organisms. The majority of those studies focused on the interaction of nanoparticles in blood vessels and the extracellular matrix [199, 200]. Interestingly, pharmacokinetics studies of metallic nanoparticles have shown a shorter blood half-life in rodents than in pigs or monkeys, an effect that should alert investigators on the use of small laboratory animals in the case of metallic NPs [200]. Their physicochemical characteristics (size, shape, aggregation, chemical composition, cellular uptake, etc.) constitute a big advantage in using these systems [201, 202], but nanotoxicity represents a limit in their extensive application [203, 204]. Researchers described apoptosis, oxidative stress, autophagy, and mitophagy as mechanisms of toxicity during nanoparticle-related uptake in in vitro [205–207] and in vivo systems [205].

*Titanium dioxide nanoparticles* (TiO_2_-NPs) are the most common nanoparticles, and they can easily cross biological barriers [208]. In vivo, toxicity of TiO_2_-NPs has been studied in microorganisms, algae, invertebrates, and vertebrates [203]. In particular, TiO_2_-NPs exert their action by causing lipid peroxidation thereby damaging membrane structures. However, once inside the cell, TiO_2_-NPs can damage organelles such as the mitochondria [203], an effect that, even if not yet proved, might promote mitophagy. Recently, the in vitro treatment of human trophoblast cells with TiO_2_-NPs caused an increase in oxidative stress and mitophagy was detected. PINK 1 and Parkin accumulated in the mitochondria and LC3-II/LC3-I, p62, and Beclin1 increased as well [207].

The same effect was observed in hepatic cells and CAL 27 cells treated with SPIO-NPs (*superparamagnetic iron oxide nanoparticles*) [209] and ZnO-NPs (*zinc oxide nanoparticles*) [210], respectively. In vitro, through the increase of ROS content, these two different nanoparticles activated PINK1 that caused mitophagy through Parkin phosphorylation. Interestingly, this study also suggested that an increase in PINK1 fluorescence can be used as a tool to assess the induction of mitophagy upon nanoparticle exposure. A connection between ZnO-NPs and mitophagy has also been demonstrated, always in vitro, in murine microglia BV-2 cells. In fact, the treatment of BV-2 cells with increasing micromolar doses of ZnO-NPs increased ROS production and the association and mitochondrial translocation of the PINK 1-Parkin complex with the induction of mitophagy. Interestingly, if PINK1 was silenced, there was no PARKIN accumulation into the mitochondria an effect that increased ZnO-NP toxicity. These results confirmed the protective role of mitophagy [211].

Another interesting nanomaterial is represented by *gold nanoparticles* (AuNPs). In non-small-cell lung cancer (NSCLC) cells, AuNPs increased TRAIL toxicity by upregulating mitochondrial fission protein DRP1 and mitochondrial fragmentation followed by mitochondrial dysfunction that cannot be reversed by mitophagy [212].

*Mesoporous silica nanoparticles* (MSNPs) may be used in biomedical applications and drug delivery to different human body areas. In endothelial cells and neurons, MSNPs with a diameter higher than 30 nm induced mitochondrial damage followed by mitophagy to remove dysfunctional mitochondria. The authors, therefore, suggested that future in vivo experiments should use MSNPs with a diameter below of 30 nm to reduce cellular toxicity [213].

In vitro studies have also been conducted on the possible correlation between *copper oxide nanoparticle* (CuONP) toxicity and hydrogen peroxide [214, 215]. The majority of ROS production is imputed to damaged mitochondria, as discussed above, and growing evidence suggests the implication of mitochondrial dysfunction in CuONP-mediated toxicity [214, 215]. It has been demonstrated that CuONPs induced anion superoxide production that, in turn, leaded to an increase in the initial steps of mitophagic flux. These copper oxide nanoparticles are located in lysosomes inside the cell where generated lysosome dysfunction inhibits mitophagy and promotes cell death [216].

Another important aspect pertains to the possibility to modify nanoparticles (NPs) so to facilitate not only cellular uptake but also their delivery to mitochondria. To this effect, NPs have been conjugated to peptides followed by immunofluorescence. Results have demonstrated that *NP-peptide conjugates* targeted mitochondria, causing membrane depolarization thereby inducing mitophagy [217]. These pilot, in vitro, experiments can help to understand the interaction between NPs and cells as well as the modifications that can increase the delivery efficacy of drugs by using NPs.

Since nanoparticles will most likely represent an important tool in future medicine, the most pressing problem concerns the little we know about their *acute* and *chronic* toxic effects on the human body [218]. This aspect has been extensively reviewed elsewhere [219, 220]. The most important aspect of NPS to consider when talking of toxicity depends on their size, composition, shape, and the large surface to mass ratio. In fact, for each type of NP, toxicity must be unraveled through in vitro and in vivo experiments. So far, a large number of in vitro experiments have been performed demonstrating how plasma membrane alteration, oxygen reactive species production, and uptake and modification of intracellular pathways cause cell damage and death. On the contrary, only a few in vivo experiments have been conducted as reviewed in [200] and [221]. On the other hand, in vivo experiments will take a considerable amount of time and high costs, and therefore, there is also a need for reliable in vitro models where to test NP toxicity [221].

Finally, in the case of mitochondria, NP effects regarding the role of autophagy and mitophagy are largely incomplete. However, a result emerging so far is that the mitophagic process observed with NPs is largely due to the NP-induced ROS accumulation that, in turn, would activate PINK 1 and/or PARKIN. A direct effect of NPs on the mitochondria to activate mitophagy might be prompted by NPs crossing the damaged plasma membrane and accumulating in the mitochondrial outer membrane or in the case of engineered NPs with mitochondria-targeted peptide.

Nonetheless, evaluating all the mechanisms that trigger nanotoxicity-mediated autophagy and mitophagy can offer a way for the toxicity assessment, for pharmacological interventions to achieve improvements for a better nanoparticle biosafety.

## 7. Conclusion

**Figure 5:**
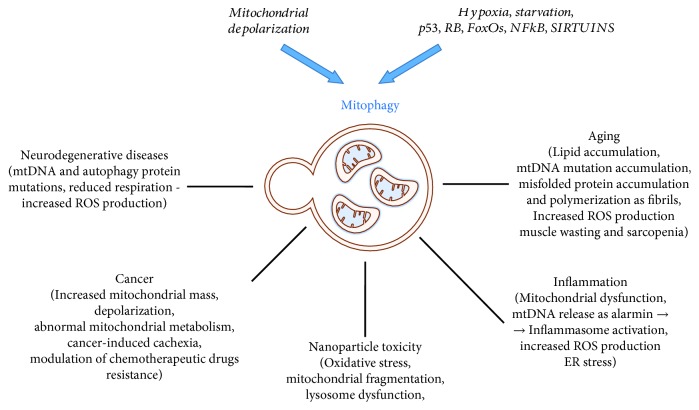
Schematic representation of all the aspects related to mitophagy. Two basic mechanisms have been elucidated involving a different set of molecules in relation to the prototype damage: the mitochondrial depolarization and hypoxia, starvation, and genes controlled by some master transcription factors (p53, RB/E2F, FoxOS, NFkB, sirtuins, and others).

The mitochondria affected by oxidative damage and aging need adequate clearance carried out by the mitophagic process coupled with mitochondrial neogenesis (growth and fission). These are essential mechanisms for the cell to adapt and respond to changing energetic requirements. Therefore, as discussed in this review, abnormal mitophagy is involved in a variety of pathologic processes such as cancer, age-related diseases, and neurodegenerative and oxidative stress disorders (Figure 5). The alteration of mitophagy has an effect related to the environment, downstream and upstream effectors, cell status, etc. In addition, the fact that nanotoxicity is mediated through autophagy and mitophagy underlines the delicate role those processes have inside the cell in determining cellular health and survival. According to the context, mitophagic changes can play a promoting or inhibitory role in tumorigenic cells triggering a cascade of effects inside the cell or acting indirectly on different complexes such as the immune system. As cancer, age-related neurodegenerative diseases remain still without an effective cure. A lot of data elucidated the connection between mitophagy and age-related illnesses and how small alterations in mitochondrial autophagy can have amplified consequences on the neurons, eyes, muscles, myocardium, and liver. Alterations in ROS production as a result of defective mitophagy can act on a broad spectrum of targets like inflammatory cytokines, hyperglycemia-related pathologies, liver ischemia/reperfusion injury, and HIF-mediated arrangements. Still a lot of work remains to do to fully know the mechanisms and the interactions behind each of them, but it seems possible that the future understanding of a variety of disease can go through the mitophagic process.

## Figures and Tables

**Table 1:** Agents affecting mitophagy.

Agent	Effect on mitophagy	Mechanism	Reference
Nicotinamide derivatives	Increase	NAD+ replenishment increases DCT-1- (ortholog to the mammalian BNIP3L/NIX) associated mitophagy in worms	[6]
Spermidine	Increase	Induces ATM activation that, in turn, promotes the accumulation of PINK1 and translocation of Parkin to mitochondria	[7]
Urolithin A	Increase	Upregulat**es** of PINK1, DCT-1, and SKN-1 Mechanism not known	[8]
Rapamycin	Increase	Increase**s** the translocation of p62 and Parkin to the damaged mitochondria	[9]
Metformin	Increase	Decrease**s** the inhibitory interaction between Parkin and p53 and increase the degradation of mitofusins	[10]
Chloroquine	Inhibition	Inhibits phagosome/lysosome fusion	[11, 12]
Mitochondrial toxins: FCCP/CCCP, rotenone, antimycin A, valmycin, oligomycin, 1-methyl-4-phenyl-1,2,3,6-tetrahydropyridine (MPTP), and 6-hydroxydopamine	Increase	Perturb mitochondrial ATP production and cause ROS generation	[13]
Ceramides	Increase	Interact directly with LC3B-II upon Drp1-dependent mitochondrial fission, leading to inhibition of mitochondrial function and oxygen consumption	[14]
Selenite	Increase	Induces superoxide-mediated mitophagic cell death	[15]

**Table 2:** Mitochondrial receptors and their ligands involved in the mitophagic process.

Receptor	Localization	Ligands (interaction)	Species	Reference
ATG32	Outer mitochondrial membrane	Atg8, Atg11	Yeast	[22]
NIX/BNIP3L	Outer mitochondrial membrane	LC3	Mammals	[23–25]
BCL2L13	Outer mitochondrial membrane	LC3	Mammals	[26]
FUNDC1	Outer mitochondrial membrane	LC3	Mammals	[27]
Cardiolipin	Mitochondrial inner membrane, any damage to mitochondria, or depolarization of its membrane results in the translocation to outer mitochondrial membrane	LC3	Mammals	[28]
PHB 2	Inner mitochondrial membrane	LC3	Mammals	[29]
Parkin	Normally in the cytosol, it is translocated to the outer mitochondrial membrane upon depolarization	AMBRA1, LC3	Mammals	[30]
BCL2L13	Outer mitochondrial membrane	LC3	Mammals	[31]
FKBP8	Outer mitochondrial membrane	LC3	Mammals	[32]
SMURF1	Cytoplasmatic, colocalized with damaged mitochondria	LC3?	Mammals	
BNIP3	Outer mitochondrial membrane	LC3	Mammals	[33]
NLRX1	Outer mitochondrial membrane	LC3	Mammals	[34]

**Table 3:** Markers of mitophagy.

Marker	Localization	Species	Reference
Aup1	Mitochondrial intermembrane space	Yeast	[2]
Uth1	Cytoplasmic leaflet of the outer mitochondrial membrane	Yeast	[3]
LGG-1	Membrane of phagophore and autophagosome	Yeast	[51]
PINK1	Normally undetectable, stabilized on the outer mitochondrial membrane when mitochondria are depolarized	Mammals	[52]
Parkin	Normally in cytosol, it is translocated to the outer mitochondrial membrane when mitochondria are depolarized	Mammals	[30]
LC3-II	Cytosolic, during autophagy, recruited to form autophagosomal membranes	Mammals	[53]
p62	Parkin recruited to mitochondria	Mammals	[54]
TOM20	Outer mitochondrial membrane	Mammals	[38]
TIM23	Inner mitochondrial membrane	Mammals	[27]
CypD (cyclophilin D)	Mitochondrial matrix	Mammals	[55]
HSP60	Mitochondrial matrix	Mammals	[55]
ULK1	Recruited to fragmented mitochondria in response to hypoxia or FCCP	Mammals	[56]
SMURF1	Cytoplasmatic, colocalized with damaged mitochondria	Mammals	[57]
Mitofusins 1/2	Mitochondrial outer membrane	Mammals	[58]
